# Childhood premorbid adjustment as a marker for 20-year impairments in schizophrenia spectrum disorder: results from the OPUS study

**DOI:** 10.1007/s00787-026-03043-1

**Published:** 2026-05-04

**Authors:** Jiasi Hao, Marie Starzer, Helene Gjervig Hansen, Carsten Hjorthøj, Merete Nordentoft, Wim Veling, Nikolai Albert, Behrooz Z. Alizadeh

**Affiliations:** 1 Department of Epidemiology, University Medical Center Groningen, University of Groningen, Hanzeplein 1, Groningen, 9713 GZ The Netherlands; 2 Mental Health Center Copenhagen, Copenhagen Research Center for Mental Health–CORE, Copenhagen University Hospital, Copenhagen, Denmark; 3 Department of Public Health, Section of Epidemiology, University of Copenhagen, Copenhagen, Denmark; 4 Department of Clinical Medicine, University of Copenhagen, Copenhagen, Denmark; 5 Department of Psychiatry, University Medical Center Groningen, University of Groningen, Groningen, The Netherlands

**Keywords:** Schizophrenia Spectrum Disorders, Premorbid, Functioning, Social, Longitudinal, Development

## Abstract

Growing evidence reveals relative stability in long-term clinical and functional outcomes of schizophrenia spectrum disorders (SSD), and links poor premorbid adjustment (PA) to worse prognoses. This study investigated associations between PA across developmental stages and long-term outcomes. Data on 496 participants (age 26.8 ± 6.5, male 58.5%) from the Danish randomised OPUS I trial were used (January 1998–December 2000; NCT00157313). Participants, with a first diagnosis within SSD, were recruited from outpatient and inpatient mental health services in Denmark and followed up for twenty years (retention = 29.0%). PA was assessed retrospectively at baseline using the Premorbid Adjustment Scale. Academic and social domain scores in childhood, and their subsequent changes in early and late adolescence, were used. Mixed models examined their effects on social functioning (Global Assessment of Functioning) and negative symptoms (Scales for the Assessment of Negative Symptoms). Low social functioning was associated with worse Social (β[95%CI] = 6.46 [0.54, 12.38], *P* ≤ 0.033) and Academic (9.40 [3.35, 15.44]; *P* ≤ 0.002) PA in childhood. Severe negative symptoms were associated with impaired Social PA in childhood (1.23 [0.73, 1.73], *P* ≤ 0.001), deterioration of Social PA in early adolescence (1.64 [0.93, 2.35], *P* ≤ 0.001), and declined Academic PA in late adolescence (0.77 [0.14, 1.39], *P* ≤ 0.016). Poor PA in childhood and PA decline during adolescence are related to Severer negative symptoms and lower social functioning 20 years after SSD onset. Our findings suggest that early signs may emerge in childhood, alongside age-dependent vulnerability-accumulation processes, when developing SSD. Support for maladjusting individuals during development is necessary to mitigate long-term impairments.

**Supplementary Information:**

The online version contains supplementary material available at https://doi.org/10.1007/s00787-026-03043-1.

## Introduction

In schizophrenia spectrum disorders (SSD), functional and clinical outcomes are essential for clinical recovery and societal reintegration. While symptom remission might be achievable for the majority of first episode psychosis patients [5, 55], the rate of clinical recovery has seemingly not improved over time [22, 40, 46, 55]. Furthermore, achieving sustained clinical recovery in the mid- to long-term still poses a considerable challenge [90], as the individuals might experience intermittent symptoms and/or relapse [88]. Nevertheless, a growing number of studies that investigate trajectories of functional and clinical outcomes, such as negative symptoms and social functioning, have unveiled a relative level of stability in the mid-to-long term [24, 25, 36, 37, 83, 92].

Meanwhile, determinants in the premorbid and prodromal stages of SSD may be potential targets for early identification and intervention in order to prevent the onset of psychosis and/or to yield a better prognosis in SSD. Commonly reported in research, these determinants include childhood adverse experiences (e.g., bullying, abuse), individual traits (e.g., personality and resilience), and living environment (e.g., neighbourhood socioeconomic status and urbanicity) [76]. Among these determinants, impaired premorbid adjustment has been found to be indicative of more adverse clinical and worse functional outcomes. This relationship was substantiated by numerous studies [42, 89], including those that adopt a life-course epidemiological viewpoint [43, 44, 92], and thereby suggest the long-lasting, continuous impacts of developmental functioning (i.e., the level of adjustment) during childhood and adolescence on overall life and illness prognosis.

A deterioration of developmental functioning is associated with an unfavourable trajectory, like persistently severe negative symptoms and compromised social functioning, in individuals later diagnosed with SSD [43, 44, 92]. Conversely, normative developmental functioning tends to align with a favourable level of outcomes over time, even in the presence of SSD. Evaluating developmental functioning during the premorbid phase, premorbid adjustment can reflect an individual’s exposure and experience during each period (i.e., childhood [up to age of 12], early adolescence [age 12–16], and late adolescence [age 16–19]). Previously, we have discussed how it may reveal the dynamic interplay of individual-environmental interactions, cognitive development, and other unaccounted determinants, each of which potentially contributes to diagnoses and their courses [43]. Therefore, the premorbid adjustment assessed at each stage may provide insight into early identification and interventions for SSD. Although substantial evidence supports the link between premorbid adjustment and clinical and functional outcomes, rigorous investigations are required to account for the temporal dynamics of premorbid adjustment and their influences on long-term outcomes. We hypothesized that premorbid adjustment could indicate the long-term post-diagnostic trajectories of social functioning and negative symptoms. In this study, we aimed to investigate the relationship between premorbid adjustment at each developmental stage and social functioning and negative symptoms during the 20 years following the first diagnosis within SSD.

## Methods

### Study population

Data from the Danish randomised OPUS I trial were used. In brief, between January 1998 and December 2000, participants were recruited from outpatient and inpatient mental health services in the central (Aarhus) and capital region (Copenhagen) of Denmark [71]. Inclusion criteria were an age of 18–45 and a first diagnosis within the schizophrenia spectrum in accordance with the International Statistical Classification of Diseases and Related Health Problems, Tenth Revision (ICD-10, codes F20–25 and F28–29) [93]. Participants were excluded if they had 1) > 12 weeks of continuous antipsychotic treatment, 2) substance-induced psychosis (ICD-10, code F1x.5), 3) IQ < 70, or 4) organic mental disorders (ICD-10, code F00–09). At inclusion, there were 578 participants, representing 90% and 63% of incident cases registered in the catchment areas of Aarhus and Copenhagen, respectively. The participants were randomized with concealed allocation sequence, 1:1, to either two years of early intervention services (EIS, OPUS intervention, *N* = 275) or mental health community treatment (standard treatment as usual [TAU] at the time, *N* = 272). A small third arm was randomly selected from the inpatient participants in EIS and TAU (1:1) to hospital-based rehabilitation (HBR, *N* = 31) [66]. This ensured that the comparison between the primary EIS and TAU arms remain balanced while allowing for the assessment of intensive inpatient population. All participants were followed up at 1, 2, 5, 10, and 20 years.

For this study, given that the present research question is independent of the initial randomization, randomization status is not considered herein, meaning that all recruited participant data were used. Specifically, the inclusion of the participants in HBR was to enhance the sample size and improve the generalizability of this study also to the people with relatively severe conditions. Next, participants with schizotypal disorders (ICD-10 code F21) were excluded due to subthreshold positive symptoms, resulting in 496 participants included. At each follow-up, the number of participants was 381 (76.8%), 336 (67.4%,), 267 (53.8%), 306 (61.7%) and 144 (29.0%), and the reasons for dropouts have been previously reported [41].

The OPUS trial was approved by the Regional Ethical Scientific Committee (Research no. KF01-387/97) and by the Danish Data Protection Agency (RHP-2017-047, I-Suite nr: 05855); it was registered at ClinicalTrials.gov (NCT00157313). All participants provided written informed consent.

### Measurements

#### Premorbid adjustment (PA)

The Premorbid Adjustment Scale (PAS) was used to assess the attainment of developmental goals in childhood (up to age of 12), early adolescence (age 12–16) and late adolescence (age 16–19) [19]. This five-item questionnaire incorporates five psychosocial domains (i.e., sociability and withdrawal, peer relationships, scholastic performance, adaptation to school and social-sexual functioning), whereas social-sexual functioning is not assessed in childhood. Following standard PAS procedures, the ratings was retrospectively obtained at baseline and reported by parents of the participants [51, 82]. Only ratings referring to the assessed age stage at least six months preceding the first onset of psychosis was included to reduce contamination by prodromal symptoms [51]. Overall, the PAS is a widely used instrument with well-documented psychometric properties, including high internal consistency, good inter-rater reliability and evidence of retrospective validity [2, 13, 52, 57].

The PAS can be calculated across age stages per domain [10, 15, 92]. We used the social (PAS-social, i.e., the mean of sociability and withdrawal, peer relationships, and social-sexual functioning) and academic (PAS-academic, i.e., the mean of scholastic performance and adaptation to school) subscales at each stage, ranging from 0 (*normal adjustment*) to 1 (*severe impairment*). To improve on the discrete subgrouping of our previously identified premorbid adjustment trajectory patterns in the presence of potential continuous heterogeneity [43, 89] and to address the potential collinearity, we also calculated and used the PAS domain change scores by subtracting the score assessed in the later stage from the one in the earlier stage [56]. For example, regarding the PAS-social domain, Δ1 = *PAS-social*_*early adolescence*_*–PAS-social*_*childhood*_, *Δ2 = PAS-social*_*late adolescence*_*–PAS-social*_*early adolescence*_. A higher score indicates worse premorbid adjustment. An increased score (Δ > 0) displays deviance from the age-appropriate level of developmental functioning, whereas a decreased score (Δ < 0) reflects a restoration to the age-appropriate level of developmental functioning.

#### Outcomes

Outcomes were rated by clinicians. Social functioning was measured by the functioning domain of the Global Assessment of Functioning (GAF) [47]. Ranging from 0 to 100, the scale was converted (i.e., *score*_*converted*_*=100–score*_*original*_) so that a higher value indicates worse social functioning, consistent with the PAS scoring. The negative symptoms were assessed and calculated using items from the Scales for the Assessment of Negative Symptoms (SANS) [3], ranging from 0 to 5, with a higher value indicating more severe negative symptoms. A detailed explanation of the calculation can be found in a previous publication [83]. Both social functioning and negative symptoms were evaluated at all follow-ups.

#### Covariates

Duration of untreated psychosis (DUP) in years was included. DUP was defined as the time from the emergence of at least one psychotic symptom to the initiation of adequate treatment. Onset and temporal course of psychotic symptoms were assessed using a modified version of the Interview for Retrospective Assessment of Onset of Schizophrenia (IRAOS) [38]. Symptom duration was recorded in monthly intervals, and symptoms were considered present if they met a threshold of at least 1 on the Present State Examination Rating Scale II, indicating that the symptom had definitely occurred during the specified period [38]. Age of onset was not entered in the models due to the consideration of multicollinearity with baseline age and years of untreated psychosis. Furthermore, available at all follow-ups, the positive symptoms were assessed by clinicians using the Assessment of Positive Symptoms (SAPS). A score was calculated from the SAPS items [4], ranging from 0 to 5, with a higher value indicating more severe positive symptoms [83].

### Statistical analyses

The baseline characteristics were summarized by mean (standard deviation [SD]), median (interquartile range [IQR]) and count (N, along with percentage). Groupwise and pairwise differences across EIS, TAU and HBR were examined (Supplementary Methods). The cross-sectional distribution and longitudinal evolution of PA and outcomes were visually inspected. Six data points (GAF_converted_=100) were excluded as outliers due to their potential influence on model estimates. Correlations between PA and outcomes were examined. Cross-sectional univariate regressions of each outcome demonstrated consistent direction and effect sizes of PA on outcomes across follow-ups, ensuring that the dropouts are not likely to introduce attrition bias to the relationship of interest. Next, univariate mixed models were applied, using the *lme4* package [11]. The fixed effects of the model were initiated with the entry of the PAS-social and its corresponding change scores (model 0, social), PAS-academic and its corresponding change scores (model 0, academic), and both domain scores and their change scores (model 1), with random intercepts of participants (i.e., to capture the between-person variations in baseline outcome levels that was not explained by the fixed effects, and controls for within-person correlation across repeated measures) and follow-up years (i.e., to capture between-year differences across the follow-ups, independent of participants, and controls for within-year observation clustering). Further adjustment included baseline age, sex (model 2), DUP, positive symptoms (model 3), and years of follow-ups in linear (model 4) or non-linear spline (model 5) functions. A natural cubic spline with two degrees of freedom and knot placement determined by the data distribution was employed [70]. This allows flexible modelling of non-linear patterns associated with symptomatic remission and relapses. All models reported no multicollinearity (variance inflation factor < 5) and satisfied underlying model assumptions (i.e., normal distribution and independence of random effects, normal distribution of residuals, and homoscedasticity) through visual inspections from the *performance* package [59]. Sensitivity analyses were conducted through re-running the models above using only participants with complete data or excluding HBR participants. Finally, considering two facts that (1) social functioning, negative symptoms and positive symptoms could affect each other [39], and (2) the results from the univariate mixed models, a Bayesian multivariate mixed model was run, using the *brms* package [16]. All model parameters were well converged, and their posterior estimates and 95% credible interval (CrI) were reported, given the data and prior information [1]. All analyses were performed in RStudio Version 4.4.3 [74].

## Results

### Participant characteristics

**Fig. 1:**
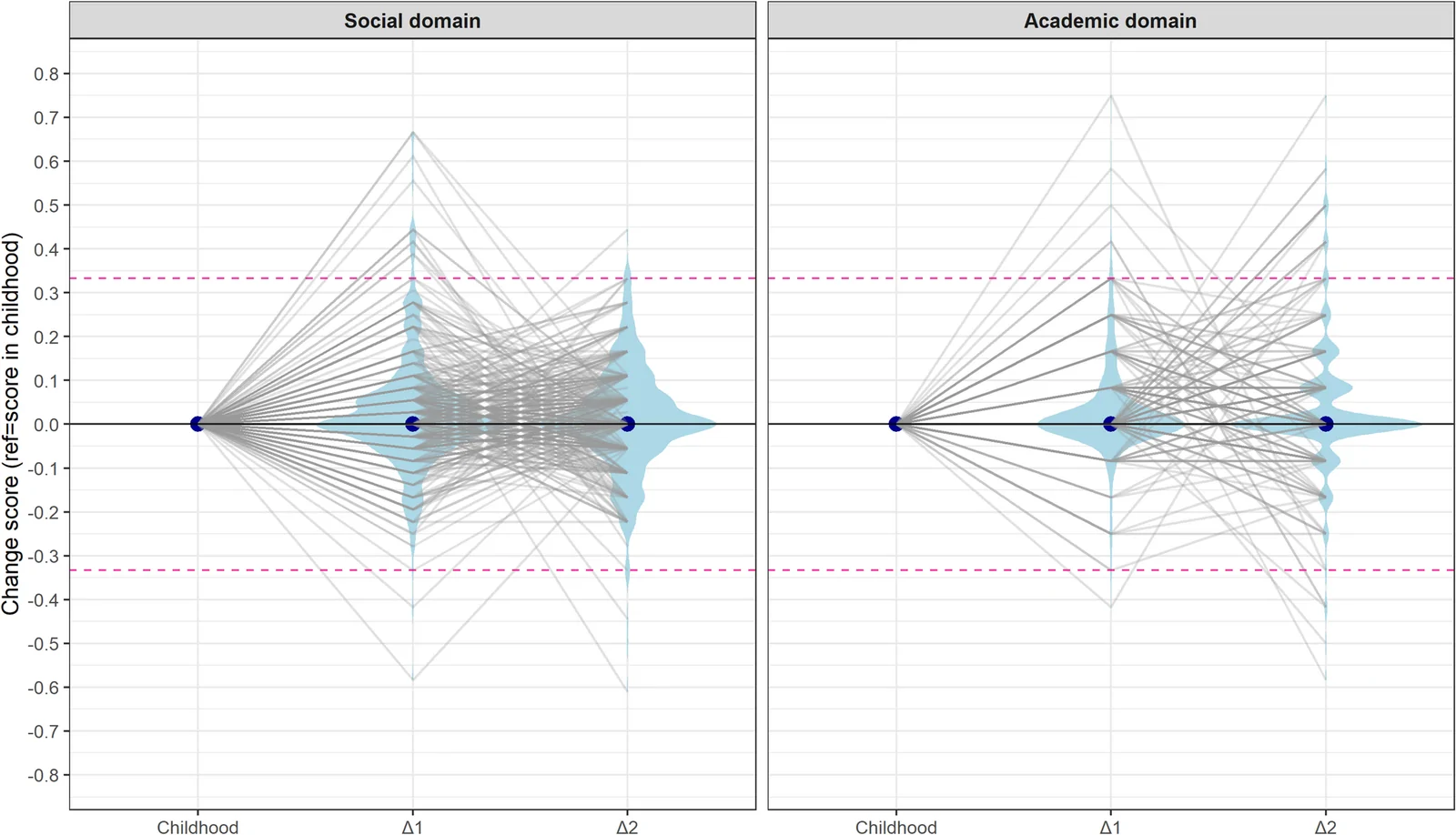
Cross-sectional distribution and longitudinal evolution of premorbid adjustment

*Note*: Premorbid adjustment was assessed by the social and academic domains in the Premorbid Adjustment Scale across childhood (age up to 12), early adolescence (age 12–16) and late adolescence (age 16–19). The values assessed in childhood serve as the reference value, Δ1 = values assessed at early adolescence minus the ones assessed in childhood, and Δ2 = values assessed at late adolescence minus the ones assessed in early adolescence. A higher value represents more deviation from achieving the age-specific developmental goal. The point at each period corresponds to the median value given the period. The pink dash lines indicate ± 0.33 (= 2/6), which is the common change cut-off of change used in publications.

Table 1 demonstrates the characteristics of the 496 participants included in this study. They were mainly young (mean age: 26.8 ± 6.5), male (58.5%), Danish (93.0%). At baseline, most participants had a low-to-medium level of education (88.1%) and were single (87.3%). The average age of first psychosis onset was 24.3 ± 6.5 with a median DUP of 1.0 [0.3, 3] year. Alongside the 20-year course of outcomes (Supplementary Fig. 1–2), the individual PAS evolvements differ across developmental stages (Fig. 1; raw stage scores in Supplementary Fig. 3). Such reconstruction of PAS stage scores addressed high correlations (Supplementary Fig. 4–5). Overall, individuals in EIS and TAU demonstrated no significant characteristic difference (Supplementary Table 1), and comparatively, participants in HBR appeared to be slightly more severe cases (Supplementary Results).

**Table 1:** Baseline characteristics of the participants included in this study

Mean (SD), Median [IQR], *N* (%)	*N* = 496
Age, year	26.8 (6.5)
Sex, male	290 (58.5)
Nationality, Danish	453 (93.0)
Education level ^a^	
Low	233 (47.6)
Medium	198 (40.5)
High	58 (11.9)
Marital status	
Single	428 (87.3)
Married	30 (6.1)
Separated or divorced	31 (6.3)
Widowed	1 (0.2)
Living arrangement	
Independent	316 (64.8)
With partner or parents	143 (29.3)
Supported living or homeless	29 (5.9)
Age of onset, year	24.3 (6.5)
Duration of untreated psychosis, year	1.0 [0.3, 3.0]
PAS, academic domain	
Childhood	0.39 (0.21)
Early adolescence	0.42 (0.22)
Late adolescence	0.44 (0.21)
Average	0.42 (0.20)
PAS, social domain	
Childhood	0.25 [0.08, 0.42]
Early adolescence	0.28 [0.11, 0.39]
Late adolescence	0.28 [0.17, 0.44]
Average	0.29 (0.21)
GAF reversed, social functioning ^b^	60.23 (13.15)
Ever attempted suicide, yes	140 (31.2)
Substance dependency, yes	144 (29.0)
Monthly use of cannabis, yes	128 (25.8)
Overall symptom severity ^c^	
Positive	3.0 [2.0, 4.0]
Negative	2.3 [1.3, 3.0]
Disorganized	0.7 [0.3, 1.7]
Psychiatric hospitalization, last year	
Not hospitalized	203 (41.7)
0–3 months	252 (51.7)
≥3 months	32 (6.6)
On social benefits, yes ^d^	231 (48.3)

^a^ The low, medium and high education level corresponds to the primary school and below (low), practical education and high school (medium), and applied university and university (high). ^b^ The scale was converted by 100-original value so that the higher the converted value is, the worse the social functioning is, which is more intuitive for interpretation. ^c^ The calculation of overall positive, negative and disorganized symptom severity was based on the SAPS and SANS, which were elaborated in the previous publication [83]. ^d^ The social benefits referred to the ones that did not apply to working or studying individuals. *Abbreviations*: *SD* standard deviation, *IQR* interquartile range, *N* number, *PAS* Premorbid Adjustment Scales, *GAF* Global Assessment of Functioning, *SAPS* Scale for the Assessment of Positive Symptoms, *SANS* Scale for the Assessment of Negative Symptoms

### Relationships between premorbid adjustment and outcomes

Table 2 shows the results from the univariate analyses. Social functioning was associated with childhood PAS-social (β[95%CI] = 6.5 [0.5, 12.4], *P* ≤ 0.033) and PAS-academic (9.4 [3.4, 15.4]; *P* ≤ 0.002) domains, sex (3.2 [1.1, 5.3], *P* ≤ 0.003), age (0.3 [0.1, 0.4], *P* ≤ 0.005) and positive symptoms (4.1 [3.5, 4.6], *P* ≤ 0.001). Negative symptoms were associated with PAS-social during childhood (1.23 [0.73, 1.73], *P* ≤ 0.001) and its change into early adolescence (i.e., Δ1; 1.64 [0.93, 2.35], *P* ≤ 0.001), PAS-academic Δ2 (i.e., change from early adolescence to late adolescence; 0.77 [0.14, 1.39], *P* ≤ 0.016), and sex (0.42 [0.24, 0.60], *P* ≤ 0.001). Results were robust across models 0–5; however, the association between negative symptoms and PAS-academic domains attenuated after adjusting for DUP and positive symptoms. Results remained unchanged in sensitivity analyses, except for the DUP and time effects (Supplementary Tables 2 and Fig. 8).

**Table 2:** Univariate associations between premorbid adjustment, social functioning and negative symptoms

Outcome	Social functioning		Negative symptoms	
	β [95%CI]	*P*	β [95%CI]	*P*
PAS, social, childhood	6.5 [0.5, 12.4]	0.033	1.23 [0.73, 1.73]	≤ 0.001
Δ1	6.8 [−1.7, 15.2]	0.118	1.64 [0.93, 2.35]	≤ 0.001
Δ2	5.3 [−4.1, 14.7]	0.267	0.28 [−0.51, 1.06]	0.492
PAS, academic, childhood	9.4 [3.4, 15.4]	0.002	0.49 [−0.02, 1.00]	0.059
Δ1	0.6 [−9.0, 10.2]	0.904	0.01 [−0.80, 0.82]	0.983
Δ2	5.4 [−2.0, 12.7]	0.155	0.77 [0.14, 1.39]	0.016
Sex, male (ref=female)	3.2 [1.1, 5.3]	0.003	0.42 [0.24, 0.60]	≤ 0.001
Age at baseline, years	0.3 [0.1, 0.4]	0.005	0.01 [−0.00, 0.03]	0.133
Untreated psychosis, years	−0.0 [−0.4, 0.4]	0.901	−0.48 [−0.86, −0.10]	0.021
Positive symptom	4.1 [3.5, 4.6]	≤ 0.001	−0.05 [−0.28, 0.18]	0.655
Follow-up year, linear	−10.2 [−19.6, −0.8]	0.037	0.01 [−0.02, 0.04]	0.563
Follow-up year, quadratic	−2.4 [−7.6, 2.7]	0.309	0.24 [0.20, 0.28]	≤ 0.001

The results are based on the model 5, and the results of models 0–4 are provided in Supplementary Table 2. β are the estimates of fixed effects from the univariate mixed effect model. Δ1 = values assessed at early adolescence minus the ones assessed in childhood, Δ2 = values assessed at late adolescence minus the ones assessed in early adolescence. *Abbreviations*: *CI* confidence interval, *PAS* Premorbid Adjustment Scales

**Fig. 2:**
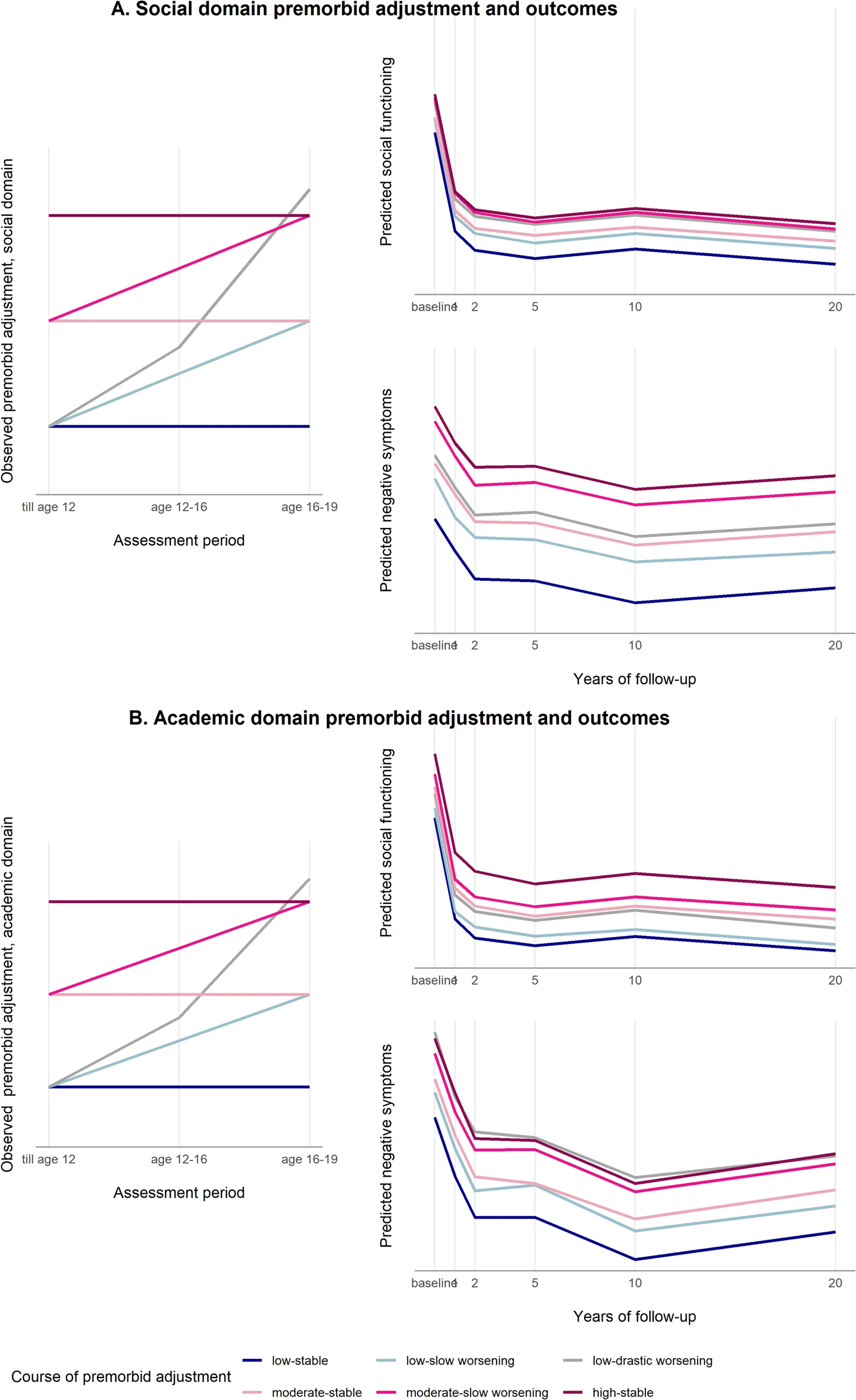
Predicted social functioning and negative symptoms based on the (**A**) social and (**B**) academic domains of premorbid adjustment

*Note*: A higher value on the y-axis in all figures represents a worse level of premorbid adjustment, social functioning and negative symptoms. The left section shows the course of the premorbid adjustment during childhood (age up to 12), early adolescence (age 12–16) and late adolescence (age 16–19) before the onset of psychosis. These courses are based on a previous publication [43]. The right section shows the levels of outcomes (i.e., social functioning and negative symptoms) over 20 years after the onset of psychosis. These levels were predicted by the multivariate model presented in Table 3 and based on each course of domain premorbid adjustment presented on the left. Overall, the figure demonstrates the association between the worse premorbid adjustment and adverse outcomes over time, with this association appearing to be moderated by the decline in premorbid adjustment.

The multivariate analysis demonstrated consistent results as described above (Table 3; Fig. 2). Additionally, social functioning was associated with changes in PAS-social from childhood into early adolescence (β[95%CrI] = 7.5 [2.2, 12.6]) and changes in PAS-academic from early to late adolescence (5.6 [1.0, 10.3]). Negative symptoms were associated with PAS-social during childhood (0.54 [0.23, 0.85]).

**Table 3:** Multivariate associations between premorbid adjustment, social functioning and negative symptoms

Outcome	Social functioning	Negative symptoms	Positive symptoms
	β [95%CrI]	β [95%CrI]	β [95%CrI]
PAS, social, childhood	7.0 [3.4, 10.6]	1.30 [1.00, 1.60]	NA
Δ1	7.5 [2.2, 12.6]	1.69 [1.26, 2.12]	NA
Δ2	3.7 [−2.2, 9.9]	0.17 [−0.32, 0.66]	NA
PAS, academic, childhood	10.3 [6.5, 13.9]	0.54 [0.23, 0.85]	NA
Δ1	−0.7 [−6.9, 5.4]	−0.04 [−0.54, 0.47]	NA
Δ2	5.6 [1.0, 10.3]	0.77 [0.39, 1.15]	NA
Sex, male (ref=female)	3.4 [2.0, 4.8]	0.45 [0.34, 0.56]	NA
Age at baseline, years	0.3 [0.2, 0.4]	0.01 [0.01, 0.02]	NA
Untreated psychosis, years	NA	NA	0.06 [0.03, 0.09]
Follow-up year, linear	NA	−0.12 [−0.25, 0.02]	−0.21 [−0.91, 0.51]

β are the estimates of fixed effects from the multivariate mixed effect model. Δ1 = values assessed at early adolescence minus the ones assessed in childhood, Δ2 = values assessed at late adolescence minus the ones assessed in early adolescence. *Abbreviations*: *CrI* credible interval, *PAS* Premorbid Adjustment Scales, *NA* not applicable

## Discussion

This study examined the relationship between the course of premorbid adjustment in childhood, early adolescence, and late adolescence in relation to long-term negative symptoms and social functioning in SSD. We found that 20 years after the first diagnosis within SSD, poor social functioning was associated with worse Social and Academic PA in childhood. More severe negative symptoms were associated with poor Social PA in childhood, further deterioration of Social PA in early adolescence compared to childhood and worsened Academic PA in late compared to early adolescence.

Substantial research has reported a robust relationship between impaired PA and poor post-diagnostic social functioning in individuals diagnosed with SSD [61]. Our findings refine this understanding by emphasizing the importance of childhood Social and Academic PA domains in shaping long-term social functioning. In the univariate analyses, subsequent developmental trajectories during adolescence, whether characterized by improvement or further decline from normative development goals, did not seem to contribute to better or worse social functioning in adulthood. This suggests that early social and academic difficulties may persist over time rather than being substantially altered by later adolescent changes, at least in relation to social functioning. One plausible explanation is low premorbid IQ. While maladjustments may stem from low premorbid IQ—a relatively stable indicator of general cognitive ability [60]—or from SSD-related brain changes influenced by factors such as injury, stress, or low IQ itself [6, 53, 64], disentangling these pathways in predisposing SSD remains challenging. Both represent established risk factors of SSD [48, 76]. However, the observed persistent impact of early social and academic difficulties may suggest the involvement of IQ in influencing social functioning [14], as one of many contributing factors within developmental vulnerability processes. It is important to note that much of this evidence is derived from schizophrenia samples, and its generalizability to the broader SSD population remains less well established. Further, the minimal score variation observed between adjacent developmental stages indicates that a minority of individuals experienced drastic deterioration in PA in our data. This observation is consistent with our prior studies and those of others reporting predominantly stable or mildly deteriorating trajectories [43, 45, 89], making it difficult to detect the expected relationship between a pronounced decline in PA and severely impaired social functioning [43, 44]. Other data sources may have more individuals exhibiting drastic deterioration [54, 81], which warrants investigation. Additionally, the Academic PA in childhood exerted a larger impact on adult social functioning than the Social PA, suggesting that it may serve as a proxy for general cognitive functioning, corresponding to the evidence that social functioning, academic performance and cognitive functioning—particularly executive and social cognition—are closely interrelated [60]. The effects of both PA domains assessed in childhood surpassed those of sex and positive symptoms, which are commonly considered supporting [39, 63, 67]. Overall, our findings potentially support a long-term stability and developmental continuity of social functioning, aligned with cumulative developmental theories and longitudinal observations in both SSD [68, 81, 92] and general populations [78, 79].

Negative symptoms demonstrate a complex relationship with development. Our results indicate that, in addition to impaired Social PA assessed in childhood, its deterioration in early adolescence was associated with more severe negative symptoms 20 years post-diagnosis. In the univariate analyses, Academic PA appeared unrelated to negative symptoms until transitioning into late adolescence, and its impact was modest compared to that of Social PA. In contrast to social functioning, negative symptoms seem to be more sensitive to developmental changes occurring across adolescence. These findings could indicate a general cumulative risk process. Individuals may begin to exhibit social difficulties in childhood, such as anxiety, externalizing (e.g., antisocial) and internalizing (e.g., withdrawal) behaviors [87]. During the transition to early adolescence, these difficulties may progressively intensify due to biological changes, including puberty and increasing social and cognitive stressors brought by role and environment changes [69]. Although these social difficulties may not immediately compromise scholastic performance or school adaptation, they could subtly exacerbate underlying neurodevelopmental vulnerabilities already at a deficit [30, 35, 49, 72]. This finding is concordant with contemporary neurodevelopmental theory in SSD, which has evolved from the earlier neurodegenerative concept of dementia praecox, to one emphasizing neurodevelopmental disruptions (e.g., delayed developmental milestones in childhood, neurological soft signs and cognitive deficiencies in early adolescence) driven by genetic, perinatal and early environmental factors [18, 28, 50, 80, 84]. Such latent disruptions could eventually contribute to suboptimal Academic PA in late adolescence when rapid cognitive maturation and demanding situations start taking place [49, 62]. The potent effects of Social PA on negative symptoms, compared to Academic PA, underscore the primacy of social developmental disruptions—such as social cognition and emotional processing that navigate the sociability, peer and close relationships, and social role fulfilling [7, 60, 65]—in the emergence and persistence of negative symptoms [33, 85]. This finding may indicate the occurrence of negative symptoms well before the onset of psychosis, which are often overlooked or misinterpreted in this prodromal phase, and therefore rarely prompt help-seeking behaviours; when getting close to the first onset of psychosis, these symptoms become overt [27]. Together with well-established sex differences [20, 21], our analysis revealed a non-linear but generally stable long-term trajectory of negative symptoms, starting with an initial improvement within the first decade post-diagnosis, followed by a gradual deterioration, consistent with earlier publications [24, 83]. However, sensitivity analyses focusing on non-HBR participants, who had better outcomes and a disappeared effect of DUP, displayed a slow and mild improving trajectory, suggesting a floor effect of DUP, even though early intervention may reduce long-term deterioration in negative symptoms [29, 32].

In the multivariate analyses, both social functioning and negative symptoms were associated with the Social PA in childhood and its transition into early adolescence and the Academic PA in childhood and its transition from early into late adolescence. The additional associations suggest shared, complex and temporal interactions of the studied factors, meriting further study. At least, congruent with current evidence [30, 35, 49, 62, 72], our study highlights the importance of recognizing early signs—some emerging as early as childhood—that require attention and support. These early signs reflect a combination of underlying neurodevelopmental, social and environmental vulnerabilities [43], potentially captured by the PAS. Early indicators may include social (cognition) impairments in theory of mind, emotion perception and social skills [12, 33, 34, 75], and neurological soft signs [9, 17, 23, 30, 34, 58], such as reduced motor coordination and sensory integration reported in comparative studies [17, 23], during childhood and adolescence. These indicators may be manifested as psychopathology when approaching the onset of psychosis in adolescence and early adulthood. Importantly, we have found that PA is associated with long-term outcomes—indicating some continuity in developmental and functional trajectories. This suggests that such a trajectory may be modified to mitigate long-term impairments, through support and interventions both following diagnosis and during earlier developmental stages (e.g., childhood as a strategic window for support and interventions).

Our study has several limitations. Data on neurodevelopmental conditions, psychiatric comorbidities, environmental adversity such as life events and traumas were not systematically available. This is important, as conditions such as autism spectrum disorders (ASD) and attention-deficit/hyperactivity disorder (ADHD) may impair premorbid functioning or share underlying liability with SSD, thereby confounding the association of PA and long-term outcomes in SSD. Similar considerations apply to childhood adversity. While the absence of such data limits inferences, it reflects the clinical heterogeneity of SSD in real-world settings, where premorbid vulnerabilities frequently overlap and are not readily disentangled, and where comorbid neurodevelopmental conditions [77, 86] and early adversity [26, 91] have been linked to poorer functional and clinical outcomes. We therefore acknowledge that part of the association between PA and outcomes may reflect broader developmental vulnerability, including underlying neurodevelopment, environmental exposures and their cumulative effects. However, disentangling these pathways was beyond the scope of the present study. In addition, our study design permits a limited causal or developmental cascading inference, yet the observed associations are consistent with a large body of literature and our previously reported causal association between PA and social functioning in the GROUP study [68]. Finally, the longitudinal nature did not exempt the study from dropouts, nevertheless, the consequent attrition bias was investigated to have minimal impacts on our findings (Supplementary Fig. 6–7).

Accumulating evidence reveals a dynamic interplay among neurodevelopment, sociodemographic determinants and life experiences, together shaping vulnerability to SSD in growing individuals. While most early signs might be nonspecific and overlap with other psychological and psychiatric conditions, future studies should embrace this ambiguity by incorporating data on comorbid traits and diagnoses. This may deepen our understanding of shared mechanisms underlying the emergence of overlapping conditions and inform early support and intervention strategies with broader preventive potential. Meanwhile, future studies should also adopt integrative, prospective approaches that examine overall development and general contextual factors holistically. This potentially helps elucidate an age-dependent process of risk aggregation and supports the translation of findings into clinical practices and policy design. Advanced modelling techniques, such as graph-based, differential equation and system models, offer promising tools to capture the evolving interactions between the biological and social factors and to facilitate causal inference, toward an overarching framework for SSD development. Lastly, evidence that similar PA patterns are observed in unaffected siblings and controls suggests that PA may index broader developmental vulnerability processes [43]. Continued development of school-, community- and family-based mental health programs [8, 31, 73] should be further refined accounting for age-specific development when providing early, timely support to children and adolescents in need.

The key strength of this study lies in its meticulous examination of how changes in PA across developmental stages influence long-term outcomes up to two decades after the onset of psychosis. By repurposing a widely used and studied instrument, we could allow for greater granularity, liberating from the constraints of latent group classifications, and meanwhile, offer novel broad insights that bridge child and adolescent developmental trajectories with the course of clinical and functional outcomes in SSD. Besides, utilizing 20-year follow-up data of a large sample of individuals affected with SSD, we could underscore the enduring influence of PA, especially in childhood, and advocate for increasing focus on early-life determinants of SSD.

Taken together, this study contributes to understanding the developmental antecedents of long-term social functioning and negative symptoms in SSD. Our findings suggest that premorbid adjustment reflects a composite developmental signal, capturing the cumulative influence of cognitive, social and environmental vulnerabilities from early life, reinforcing the central role of early support and interventions.

## Conclusion

Impaired premorbid adjustment in childhood or decline in premorbid adjustment during adolescence may have a persistent, negative impact on long-term negative symptoms and social functioning in people diagnosed with SSD. These results highlight the importance of early developmental vulnerability and suggest an age-dependent risk process in SSD. Current early support for those children and adolescents exhibiting maladjustments should be further refined to the evolving development and their application may be crucial in mitigating long-term impairments.

## Supplementary Information

Below is the link to the electronic supplementary material.

Supplementary Material 1 (XLSX 11.0 MB)

Supplementary Material 2 (DOCX 1.66 MB)

## Data Availability

Due to privacy, we are not permitted to share the data. Researchers interested in the data may contact Marie Starzer at [marie.starzer@regionh.dk](mailto: marie.starzer@regionh.dk).
