# Supplementary material for: Childhood premorbid adjustment as a marker for 20-year impairments in schizophrenia spectrum disorder: results from the OPUS study
Source: Eur Child Adolesc Psychiatry. 2026 May 4;35(8):2645–57. doi: 10.1007/s00787-026-03043-1 (PMC13652873; doi:10.1007/s00787-026-03043-1)
Supplement: Supplementary file 1 [file 787_2026_3043_MOESM2_ESM.docx]

# SUPPLEMENTARY INFORMATION

**Childhood premorbid adjustment as a marker for 20-year impairments in schizophrenia spectrum disorder: Results from the OPUS study**

**Running title**: Childhood premorbid adjustment in 20-year impairments

J. Hao *et al*

**CONTENT**

[SUPPLEMENTARY METHODS	2](#_Toc209346174)

[Groupwise and pairwise examinations	2](#_Toc209346175)

[Visual inspections on key variables	2](#_Toc209346176)

[Correlations of premorbid adjustments by domain and developmental stage	4](#_Toc209346177)

[Investigations on dropouts and non-dropouts	5](#_Toc209346178)

[SUPPLEMENTARY RESULTS	6](#_Toc209346179)

[Baseline characteristics differences between HBR and non-HBR	6](#_Toc209346180)

[Negative symptoms over 20 years in the univariate model	6](#_Toc209346181)

[REFERENCES	7](#_Toc209346182)

## SUPPLEMENTARY METHODS

Groupwise and pairwise examinations

Groupwise differences across EIS, TAU and HBR were examined by one-way analysis of variance (ANOVA; if non-normally distributed, Kruskal-Wallis tests) for continuous variables and χ2 tests (Fisher’s exact test if cell counts <5) for categorical variables[2]. In the presence of groupwise difference (*P*<0.05), post-hoc analyses were conducted to examine pairwise differences using the Benjamini-Hochberg correction that controls the false discovery rate in multiple testing[1, 3].

Visual inspections on key variables

##### Supplementary Figure 1 Cross-sectional distribution and longitudinal evolution of social functioning.

Social functioning was assessed by the functional domain of the Global Assessment of Functioning (GAF). Ranging from 0 to 100, the scale was converted (i.e., *score_converted_=100–score_original_*) so that a higher value indicates worse social functioning. The point at each follow-up year correspond to the median value given the follow-up.

##### Supplementary Figure 2 Cross-sectional distribution and longitudinal evolution of negative symptoms.

Negative symptoms were assessed by the Scales for the Assessment of Negative Symptoms. A higher value represents worse negative symptoms. The point at each follow-up year correspond to the median value given the follow-up.

##### Supplementary Figure 3 Cross-sectional distribution and longitudinal evolution of premorbid adjustment.

Premorbid adjustment was assessed by the social and academic domains in the Premorbid Adjustment Scale across childhood, early adolescence and late adolescence. A higher value represents worse premorbid adjustments. The point at each period correspond to the median value given the period.

Correlations of premorbid adjustments by domain and developmental stage

##### Supplementary Figure 4 Correlation matrix of scores related to premorbid adjustment.

Premorbid adjustment was assessed by the social and academic domains in the Premorbid Adjustment Scale across childhood, early adolescence and late adolescence.

##### Supplementary Figure 5 Correlation matrix of premorbid adjustment in childhood and its changes.

Premorbid adjustment was assessed by the social and academic domains in the Premorbid Adjustment Scale across childhood, early adolescence and late adolescence. Δ1=values assessed at early adolescence minus by the ones assessed in childhood, Δ2=values assessed at late adolescence minus by the ones assessed in early adolescence.

Investigations on dropouts and non-dropouts

##### Supplementary Figure 6 Cross-sectional estimated effects of premorbid adjustment on social functioning and corresponding number of dropouts.

##### Supplementary Figure 7 Cross-sectional estimated effects of premorbid adjustment on negative symptoms and corresponding number of dropouts.

## SUPPLEMENTARY RESULTS

Baseline characteristics differences between HBR and non-HBR

Comparatively, the participants in HBR had earlier onset (21.1±4.8 vs. EIS: 24.7±6.2, *P*=0.019; TAU: 24.2±6.8, *P*=0.027), longer DUP (2.1 [0.9, 4.1] vs. 0.8 [0.2, 2.5], *P*=0.019; 1.0 [0.3, 3.7], *P*=0.073), higher rate of substance dependency (53.6% vs. 27.5%, *P*=0.010; 27.7%, *P*=0.010) and monthly cannabis use (50.0% vs. 27.0%, *P*=0.023; 21.7%, *P*=0.006). They had severer positive symptoms (4.0 [2.9, 4.6] vs. 3.0 [2.0, 4.0], *P*=0.021; 3.0 [2.0, 4.0], *P*=0.002), longer hospitalization time (over 3 months, 17.9% vs. 6.1%, *P*=0.001; 5.7%, *P*=0.001) and more likely to receive social benefits (81.5% vs. 44.2%, *P*=0.010; 48.4%, *P*=0.009) and to be in supported living facilities or homeless (25.0% vs. 5.2%, *P*=0.001; 4.4%, *P*=0.001).

Negative symptoms over 20 years in the univariate model

##### Supplementary Figure 8 Predicted 20-year trajectory by sex and follow-up years.

## REFERENCES

1.	Benjamini Y, Hochberg Y (1995) Controlling the false discovery rate: a practical and powerful approach to multiple testing. Journal of the Royal statistical society: series B (Methodological) 57:289-300

2.	Kazuki Y, Alexander B, Jonathan JC, Justin B, Lucy DAM, Malcolm B, Rune Haubo BC, gbouzill (2022) tableone. In:

3.	McDonald JH (2014) Handbook of Biological Statistics. Sparky House Publishing, Baltimore, Maryland
